# Multifocal pupillographic objective perimetry for assessment of early diabetic retinopathy and generalised diabetes-related tissue injury in persons with type 1 diabetes

**DOI:** 10.1186/s12886-022-02382-2

**Published:** 2022-04-13

**Authors:** Faran Sabeti, Corinne F. Carle, Christopher J. Nolan, Alicia J. Jenkins, Andrew C. James, Lauren Baker, Caitlin E. Coombes, Veronica Cheung, Melody Chiou, Ted Maddess

**Affiliations:** 1 Eccles Institute for Neuroscience, The John Curtin School of Medical Research, The Australian National University, Canberra, ACT 2601 Australia; 2 Discipline of Optometry, Faculty of Health, University of Canberra, Canberra, ACT Australia; 3 Australian National University Medical School, The Australian National University, Canberra, ACT Australia; 4 Division of Immunity, Inflammation and Infection, The John Curtin School of Medical Research, The Australian National University, Canberra, ACT Australia; 5 Department of Endocrinology, The Canberra Hospital, Canberra, ACT Australia; 6 University of Sydney, NHMRC Clinical Trials Centre, Camperdown, Sydney, NSW Australia

**Keywords:** Type 1 diabetes, Objective perimetry, Tissue injury, Metabolic variables, Pupillometry

## Abstract

**Background:**

To examine the potential utility of five multifocal pupillographic objective perimetry (mfPOP) protocols, in the assessment of early diabetic retinopathy (DR) and generalised diabetes-related tissue injury in subjects with type 1 diabetes (T1D).

**Methods:**

Twenty-five T1D subjects (age 41.8 ± 12.1 (SD) years, 13 male) with either no DR (*n* = 13) or non-proliferative DR (*n* = 12), and 23 age and gender-matched control subjects (age 39.7 ± 12.9 years, 9 male) were examined by mfPOP using five different stimulus methods differing in visual field eccentricity (central 30° and 60°), and colour (blue, yellow or green test-stimuli presented on, respectively, a blue, yellow or red background), each assessing 44 test-locations per eye. In the T1D subjects, we assessed 16 metabolic status and diabetes complications variables. These were summarised as three principal component analysis (PCA) factors. DR severity was assessed using Early Treatment of Diabetic Retinopathy Study (ETDRS) scores. Area under the curve (AUC) from receiver operator characteristic analyses quantified the diagnostic power of mfPOP response sensitivity and delay deviations for differentiating: (i) T1D subjects from control subjects, (ii) T1D subjects according to three levels of the identified PCA-factors from control subjects, and (iii) TID subjects with from those without non-proliferative DR.

**Results:**

The two largest PCA-factors describing the T1D subjects were associated with metabolic variables (e.g. body mass index, HbA1c), and tissue-injury variables (e.g. serum creatinine, vibration perception). Linear models showed that mfPOP per-region response delays were more strongly associated than sensitivities with the metabolic PCA-factor and ETDRS scores. Combined mfPOP amplitude and delay measures produced AUCs of 90.4 ± 8.9% (mean ± SE) for discriminating T1D subjects with DR from control subjects, and T1D subjects with DR from those without of 85.9 ± 8.8%. The yellow and green stimuli performed better than blue on most measures.

**Conclusions/interpretation:**

In T1D subjects, mfPOP testing was able to identify localised visual field functional abnormalities (retinal/neural reflex) in the absence or presence of mild DR. mfPOP responses were also associated with T1D metabolic status, but less so with early stages of non-ophthalmic diabetes complications.

## Background

The chronic microvascular complications of type 1 diabetes (T1D), including diabetic retinopathy (DR), nephropathy and neuropathy, contribute profoundly to the burden of this disease [1–4]. In order to assess the subclinical stages of these complications, and the effects of new therapies for their prevention, more sensitive tests for detecting both organ-specific changes such as DR, as well as generalized diabetes-related tissue injury (e.g. from widespread hyperglycemia-induced tissue damage from accumulation of advanced glycation end products and oxidative stress), are needed.

Taking diabetic eye disease as an example, the traditional treatments of laser photocoagulation, anti-vascular endothelial growth factor injections and vitrectomy, reduce the risk of vision loss, but they target late disease stages such as proliferative DR and diabetic macular oedema. These late treatments are also associated with significant side effects [5–8]. Candesartan shows promise in the prevention of earlier stage retinopathy in T2D patients [9]. Fenofibrate is also gaining recognition as a therapy with potential to prevent progression and even reverse earlier stages of DR in T2D [10, 11]. Clearly, additional new treatments that target earlier stages of diabetic eye disease are needed and these will need monitoring tools for pre-clinical early-stage disease.

Multifocal pupillographic objective perimetry (mfPOP) assesses visual function by monitoring pupil responses to retinal stimuli presented to 44 retinal locations/eye. Both response sensitivity and delay are obtained at each location of both visual fields concurrently. The method is rapid, objective and requires minimal operator training. We have shown mfPOP to be clinically useful in early [12, 13] and later stage [14–16] age-related macular degeneration (AMD). We have also shown that mfPOP is able to identify localised visual field dysfunction in type 2 diabetes (T2D) subjects prior to the development of clinically detectable retinal vasculopathy [17, 18] and early-stage diabetic macular oedema [19]. Diabetic changes to the visual fields are similar in multifocal visual evoked potentials (mfVEPs) and mfPOP when tested in the same subjects [20]. The main advantage of mfPOP is the rapid, non-invasive nature of assessment.

Given those robust outcomes, we have hypothesised that mfPOP has potential to monitor early stage diabetes-related retinal and nerve dysfunction and, in addition might be useful as a clinical test to assess more generalised diabetes-related tissue injury, that may be superior to other measures such as skin advanced glycation end product (AGE) accumulation and the urine albumin excretion rate. In these ways, it could prove to be a useful clinical tool to assess effectiveness of interventions to prevent diabetes complications in their early stages of development.

In this pilot study, we have examined the potential utility of mfPOP in the assessment of early diabetic retinopathy (DR) and generalised diabetes-related tissue injury (GDTI) in subjects with type 1 diabetes. We compare diagnostic power of five novel mfPOP stimulus methods, which test either the peripheral or macular visual fields, with parameters of metabolic status, retinopathy using the Early Treatment of Diabetic Retinopathy Study (ETDRS) scales for macular and peripheral DR, and various assessments of non-ocular diabetic complications.

## Methods

### Subjects

In this prospective study we recruited twenty-five T1D subjects with variable duration of diabetes with either no DR or non-proliferative DR, all non-smokers without other known diabetic complications from The Canberra Hospital Diabetes Clinic. Given past published results [17, 18], the required sample size for *p* = 0.05 and power of 0.8 was 10. Twenty-three age and gender matched non-diabetic control subjects were recruited from the general community. Controls were excluded if they had first-degree relatives with diabetes, previous gestational diabetes, or were smokers. T1D and control subjects were excluded if they were pregnant, had visual acuity worse than 6/12, past eye surgery, intraocular pressure > 21 mmHg, distance refraction ≥ ± 5 D or ≥ ±2 D cylinder, or had medications or comorbidities that would affect their pupillary responses.

### Non-ocular clinical and biochemical assessments

On the first of 2 visits, T1D subject weight and height was measured to calculate body mass index (BMI). Standing systolic and diastolic blood pressure was measured (average of 2nd and 3rd of 3 readings). Venous blood (sodium fluoride and serum tubes) was taken from the T1D subjects before each mfPOP session to determine plasma glucose and serum potassium concentration levels performed within the clinical laboratories of ACT Pathology at The Canberra Hospital. The most recent measures of the T1D subjects’ HbA1c, serum creatinine, triglyceride, total cholesterol, high density lipoprotein (HDL) cholesterol and low density lipoprotein (LDL) cholesterol, urinary albumin creatinine ratio, as well as all available HbA1c measures over the previous 5 years, were recorded from the subjects’ medical records. Peripheral neuropathy was assessed using a biothesiometer (Bio-Medical Instrument Company, Newbury, Ohio, USA). Skin advanced glycated end-products (AGE) were assessed by an AGE reader (DiagnOptics, Gronungen, The Netherlands).

### *mfPOP* stimuli and data acquisition

mfPOP testing was performed using a prototype of the Federal Drug Administration cleared objectiveField Analyzer (OFA) (Konan Medical USA Inc., Irvine CA) in all subjects. The OFA presented multifocal stimuli simultaneously to the two eyes via two liquid-crystal displays operating at 60 frames/s. Subjects fixated on a red cross centred within a dim starburst radial grating to aid binocular fusion. Trial lenses corrected the subject’s distance prescription and stimuli were presented at optical infinity. The mfPOP method had several quality control steps. First of all, the operator could see an image of the two fitted pupil diameters in real time, and its colour indicated quality. Poor quality would lead to a check of focus. Also a time-varying bar whose length indicated the quality of the diameter fit in real time was displayed. Blinks were monitored and data removed from the pupil record when blinks occurred. Each mfPOP test, or protocol, presented 9 test segments of 42.4 s duration (6.37 min total), with a short rest break in between segments. If more than 15% of the data from a segment was lost due to blinks it was repeated. The per-region responses were estimated by a regressive method, and that provided both t-statistics for each sensitivity and delay, and an overall goodness of fit statistic (r2) for each pupil.

**Fig. 1:**
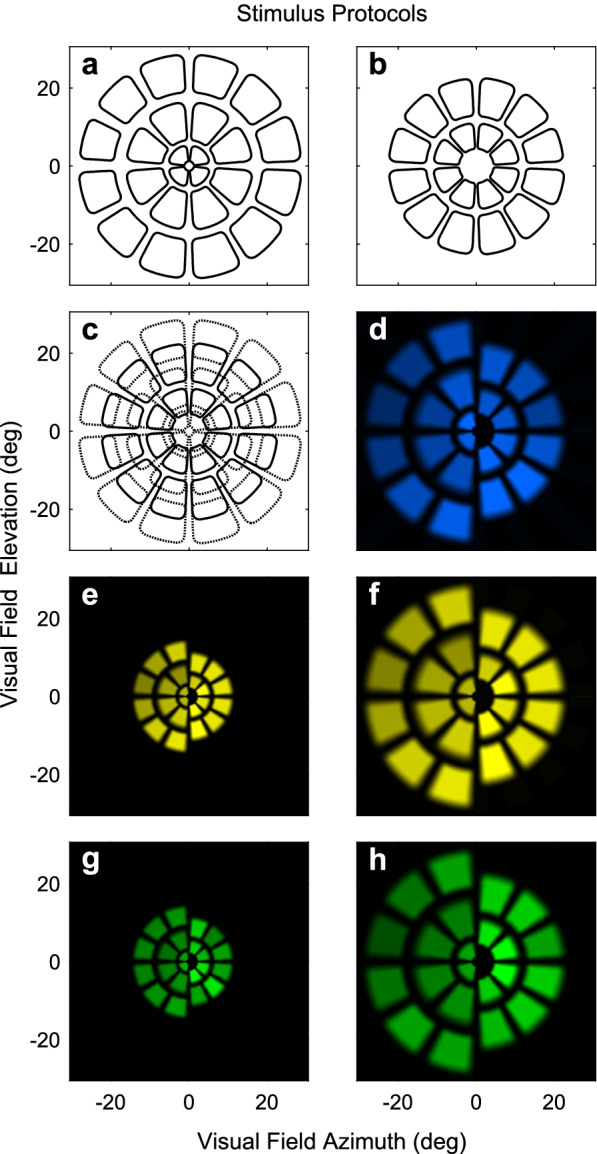
The mfPOP stimulus protocols. All 5 protocols had 44 stimulus regions arranged in 5 rings, **a** and **b**. Pseudo-random sequences governed when any one stimulus region would be shown for 33 ms. Each region was presented 96 times. The presentation sequence meant that although the stimuli could overlap (**c**) in practice they never did. a to c show the layout of stimuli for the wide-field stimuli d,f,h. The layout of the macular stimuli, e,g, was the same, just isomorphically scaled by a factor of two. In d to h left and right halves of the 2- and 3-ring stimulus arrays are presented separately to aid visibility. **d**) The Blue Wide-field stimulus, **e**) Yellow Macular, **f**) Yellow Wide-field, **g**) red-green (RG) Macular, **h**) RG Wide-field. Different parts of the fields of normal persons differ in sensitivity, therefore individual regions varied in intensity to try to balance the size of the responses from each region

Five stimulus protocols were tested in a randomised order, over two sessions, 2 weeks apart. The protocols differed in luminance, colour and visual extent (Fig. 1). Two of the protocols presented 44 stimuli/eye to the macular region (±15° eccentricity) that were either: yellow stimuli on a yellow background (*Yellow Macula*), or green stimuli on a red background (*RG Macula*). The other three protocols were wide-field methods that presented 44 stimuli/eye to a four-fold greater area (±30°). These presented yellow (*Yellow Wide*) or blue stimuli (*Blue Wide*), on the same colour background, or green stimuli on a red background (*RG Wide*).

Pseudo-random trains of transient onset stimuli (33 ms duration) appeared at a mean interval of 4 s per stimulus-region. There were 44 stimulus-regions per eye (Fig. 1), providing 22 stimuli/s in total. Each region was tested 96 times. Pupil responses were measured at 60/s under infrared illumination using two video cameras. Fixation was monitored online and data obtained during blinks or fixation losses deleted. Segments with less than 85% of their data were repeated. The luminance of each stimulus for each protocol varied depending on its location in the visual field (Table 1, ESM). This luminance balancing method improves signal quality and has been described elsewhere [21].

**Table 1:** Characteristics of control and type 1 diabetes subjects, by retinopathy status

	Characteristics	Control	No retinopathy	Retinopathy	*P*-value
	N	23	12	13	
	Age (years)	39.2 ± 13.8	44.8 ± 14.9	41.9 ± 11.9	0.668
	Fraction male	0.52	0.5	0.4	0.962
	**Ocular measures**				
	ETDRS score ranges	L 10	L 10	L 20 – L 43	
	ETDRS_cen_	L10	L10	L 20 – L 43	
	HFA mean deviation (dB)	−0.3 ± 0.9	−1.0 ± 1.4	−1.4 ± 1.1	0.361
	HFA pattern standard deviation (dB)	1.4 ± 0.3	1.4 ± 0.3	1.7 ± 0.7	0.202
	**Patient Variables**				
1	Duration of diabetes	–	23.3 ± 12	24.7 ± 10.1	0.638
2	BMI (kg/m^2^)	–	24.6 ± 2.9	29.0 ± 6.3	0.056
3	HbA1c (mmol/mol) most recent	–	65 ± 7	67 ± 6	0.431
	HbA1c (%) most recent		8.1 ± 0.9	8.3 ± 0.8	0.431
4	HbA1c (mmol/mol) 1 year ago	–	64 ± 6	70 ± 6	0.109
	HbA1c (%) 1 year ago		8.0 ± 0.8	8.6 ± 0.8	0.109
5	HbA1c (mmol/mol) 5-year mean	–	57 ± 4	65 ± 6	**0.013**
	HbA1c (%) 5-year mean		7.4 ± 0.5	8.1 ± 0.7	**0.013**
6	Total cholesterol (mmol/l)	–	4.5 ± 0.6	4.6 ± 0.5	0.590
7	Triglycerides (mmol/l)	–	0.7 ± 0.3	1.1 ± 0.4	**0.017**
8	HDL-cholesterol (mmol/l)	–	1.6 ± 0.3	1.4 ± 0.2	0.108
9	LDL-cholesterol (mmol/l)	–	2.5 ± 0.6	2.6 ± 0.5	0.539
10	AGE score	–	2.1 ± 0.4	2.3 ± 0.6	0.538
11	Creatinine (μmol/l)	–	73.9 ± 9.3	79.1 ± 12.8	0.249
12	eGFR (mL/min/1.73 m^2^)	–	85.2 ± 8.6	78.9 ± 13.3	0.164
13	UAlbCr (mg/mmol)	–	0.60 ± 0.4	1.6 ± 2.2	0.121
14	Average biothesiometer score (left and right)	–	6.7 ± 7.9	6.1 ± 8.9	0.330
15	Visit 1 Plasma glucose (mmol/l)	–	9.8 ± 3.3	11.4 ± 4.8	0.305
	Visit 2 Plasma glucose (mmol/l)	–	9.9 ± 5.1	9.6 ± 3.4	0.768
16	Visit 1 Potassium (mmol/l)	–	3.9 ± 0.3	4.0 ± 0.3	0.349
	Visit 2 Potassium (mmol/l)	–	3.9 ± 0.2	4.0 ± 0.2	0.224

Bolding indicates significant difference (*p* < 0.05) between the diabetic retinopathy groups

*AGE* advanced glycation end-product, *HFA* Humphrey Field Analyzer, *UAlbCr* urine albumin creatinine ratio

The average response waveforms for each test region were extracted from raw pupillary responses by multiple linear regression [22]. Pupil contractions were scaled by the mean pupil size over the protocol period and standardised to 3.5 mm and then transformed to decibel (dB) sensitivities [17, 22]. The times-to-peak contraction (delays) were expressed in milliseconds. With recording of both direct and consensual pupil responses to the visual stimuli to each of 44 regions of the retina of each eye, a total of 176 pairs of averaged sensitivities and delay measures was available for analysis [17, 22] for each subject.

### Additional eye assessments

On one of the two visits, subjects had 24–2 SITA Fast perimetry (Humphrey Field Analyzer (HFA); Carl Zeiss Meditec, Inc., Dublin, CA) performed. Additionally, their pupils were dilated following mfPOP testing and five 45° fundus images/eye (CR-2 Retinal Camera, Canon Inc., Tokyo, Japan) were acquired equivalent to the seven 30° photos of the Early Treatment Diabetic Retinopathy study (ETDRS) [23]. ETDRS scores were determined by the Retinal Vascular Imaging Centre (East Melbourne, Vic). Classification of DR was according to the ETDRS guidelines, were recorded at baseline and ranged between L10 to L43 (Table 1) [24]. The reading centre also provided central scores (ETDRS_cen_) using the central 45° fundus image, which corresponded more closely to the mfPOP stimulus area. We subsequently categorised the participants according to their grade of ETDRS score across both the ETDRS area and the central ETDRS area. This resulted in three severity categories (Table 1), severity 1 for ETDRS and ETDRScen corresponding to both eyes having a normal appearing fundus. Severity 2 representing T1D eyes with ETDRS and ETDRScen grading of no retinopathy and Severity 3 represents eyes with retinopathy. For the PCA factors the scores (how strong each factor was in each subject) were split into three groups according to the 33rd and 66th percentiles of the particular scores. A priori we did not know if positive or negative scores were associated with diabetic eye damage so were compared ROC analysis for positive and negative versions of the scores, and then selected the version that agreed with other disease severity markers for further analysis.

### Statistical analysis

Was performed using MATLAB (2016b Mathworks Inc., Natick, MA). An objective of this study was to examine correlation between mfPOP performance and 16 T1D subject variables (Table 1), reduced to a small number of independent uncorrelated factors using principal components analysis that describe the cohort. Details of the method are given elsewhere [25]. We used multiple regression-linear models to examine the extent to which pupillary response amplitudes and delays, adjusted for age and gender, predicted the PCA factor scores, ETDRS, and ETDRS_cen_ scores. Receiver Operator Characteristic (ROC) analysis quantified the diagnostic power of the stimulus protocols as the percent area under the ROC curves (AUCs). The ROC analysis utilized deviations from control values for the 44 times to peak and sensitivities/eye, and also scores based upon combined delay and sensitivity using our published method from our earlier diabetes study [17]. The combined scores are linear combinations of the per-region sensitivities and delays differences from normal. Additional ROC analyses were also computed for three disease severity ratings based upon ETDRS, ETDRS_cen_, and the PCA factor scores. Each of the severity rating levels was adjusted to include about equal numbers of subjects or eyes as appropriate.

## Results

### Subject characteristics

Table 1 presents the control and T1D subject characteristics including demographics, ocular assessments other than mfPOP, and metabolic and diabetes complication assessments. The table segregates those with T1D patients into those without retinopathy (*n* = 13) and with retinopathy (*n* = 12) according to the ETDRS score (≤10 or > 10, respectively). The control and T1D subjects were well matched for age and gender. Overall, the T1D subjects had long-standing diabetes (24.0 ± 11.1 years; range, 7 to 46 years) with moderate diabetes control reflected in their mean HbA1c levels of the previous 5 years (61 ± 6 mmol/mol; 7.7 ± 0.7%). One T1D subject had an estimated glomerular filtration rate of < 60 ml/min (eGFR 57 ml/min) and one had microalbuminuria (urinary albumin creatinine ratio 8.0 mg/mmol), with all others having no clinically significant nephropathy. Five T1D subjects had evidence of peripheral neuropathy (biothesiometer score > 10), and 13 subjects had evidence of mild non-proliferative retinopathy (ETDRS score of > 10–43) in at least one eye. Correlation between patient ETDRS and ETDRS_cen_ scores was moderate at 0.64 (*p* < 0.01). Of the T1D subject variables, only the 5-year mean HbA1c levels and serum triglyceride concentrations were significantly higher in those subjects with compared to those without DR. Of note, BMI was also higher in subjects with DR, but this did not reach statistical significance (*p* = 0.056).

### Prediction of retinopathy - regression modelling

We first explored which of the 16 patient variables might predict retinopathy. A stepwise regression model fitting DR vs no DR, based on the ETDRS scores, selected BMI as the only significant predictor (*p* = 0.014, F_1,23_ = 7.03). A similar model regressing upon the ETDRS_cen_ data selected serum creatinine and the 5-year HbA1c mean as significant predictors (model *p* = 0.005, F_2,22_ = 6.73). While these models indicated that some patient variables were important, stepwise regression can be unreliable [26]. To gain further insight we decided to use PCA based factor analysis to find a small number of factors within the patient variables that explain much of the variance in those measures.

### Metabolic, tissue-injury and lipid PCA factors

The largest three PCA factors explained 59% of the variance in the 16 T1D subject variables. We correlated the scores from those 3 factors with the original T1D subject variables. The upper part of Table 2 shows the 9 T1D subject variables that had the highest correlations, and in bold those with the greatest factor loadings. PCA factor 1, which explained 23.8% of the variance, was most associated with HbA1c levels, BMI and triglyceride levels, such that we refer to it as the “*Metabolic”* variable based factor (Table 2, left 2 columns). PCA factor 2, explained 20.9% of the variance, was most associated with renal function, peripheral neuropathy, and AGE scores, as well as diabetes duration, and we refer to it as the “*Tissue-injury*” factor (Table 2, central 2 columns). PCA factor 3, explained 14.5% of the variance, and was associated with total cholesterol and low-density lipoprotein cholesterol, and so we refer to it as the *“Lipid”* factor (Table 2, right 2 columns). While eGFR is significantly correlated with each of the three PCA factors, the respective factor loadings for eGFR (possible range ± 100%) were 43.4% with PCA factor 1, 49.6% with factor 2 and 6.3% with factor 3; therefore eGFR was contributing mostly to PCA factor 2, the Tissue-injury factor. Only the nine patient variables most correlated with the PCA factors are presented in Table 2, as from 10 onward, both the correlation strengths and factor loadings dropped markedly.

**Table 2:** Candidate independent PCA factors of metabolic control/tissue-injury

PCA factor 1 (Metabolic)		PCA factor 2 (Tissue-injury)		PCA factor 3 (Lipid)	
Variable	Correlation	Variable	Correlation	Variable	Correlation
BMI	− 0.73^*^	AGE	− 0.77^*^	Creatinine	0.77^*^
HbA1c 5-year	−0.65^*^	eGFR	0.76^*^	eGFR	−0.65^*^
HbA1c 2011	−0.65^*^	Biothesiometer	−0.68^*^	Cholesterol	−0.63^*^
Creatinine	−0.65^*^	Creatinine	−0.63^*^	LDL-chol	−0.62^*^
HbA1c test day	−0.62^*^	T1D duration	−0.59^*^	BMI	0.41
eGFR	0.61^*^	BMI	−0.44	T1D duration	−0.35
Triglycerides	**−0.47**	Triglycerides	−0.34	Potassium	−0.30
HDL-chol	0.43	Glucose	**0.33**	HbA1c on day	0.26
LDL-chol	0.40	Potassium	**0.31**	HDL-chol	−0.25
ETDRS	0.691^*^		0.554^*^		0.349
ETDRS_cen_	0.678^*^		0.509^*^		0.455^*^

Upper nine rows are correlation coefficients between the patient variables and the respective principal components analysis (PCA) factors. * indicates significant correlation (*p* < 0.02), bolding indicates the patient variables with the largest loadings for the respective PCA factor (i.e. the variable contributes more to that PCA factor than other PCA factors)

Lower two rows are correlations between the two types of ETDRS scores and the respective PCA factors. * indicates significant correlation (*p* < 0.02)

*AGE* advanced glycation end products

Potassium and Glucose were the means for the two visits

### Association of PCA factors with retinopathy

The lower part of Table 2 shows the correlations between the mean between-eye ETDRS and ETDRS_cen_ scores, and the Metabolic, Tissue-injury and Lipid factor scores. Of note, the correlations between both ETDRS scores and the Metabolic and Tissue-injury factor scores were at least moderate, with the ETDRS_cen_ score also being significantly associated with the Lipid factor.

### Associations of mfPOP response delays and sensitivities with retinopathy and PCA factors

The results of multiple linear regression models are presented for response delays (Table 3) and response sensitivity (Table 4). For these models we split the values of the ETDRS and ETDRS_cen_ ratings, and the three types of PCA factor scores, into three severity categories (Methods). This was done to characterise the effects of disease severity as measured by those methods. Only models containing ETDRS, ETDRS_cen_, and the Metabolic PCA factor showed strong associations with mfPOP delays (Table 3). The models were referenced to normal males (top row for each model). Age effects were non-significant and so are not shown. Females had shorter times to peak. The occasional non-significant results of the disease severity ratings are in bold, all the others were significant (*p* < 0.05). The goodness of fit (R^2^) increased somewhat from the ETDRS model through to the Metabolic factor, rising from 0.359 to 0.420. Delays increased more uniformly with increasing ETDRS_cen_ and Metabolic severity ratings. The only significant results for the Tissue-Damage and Lipid factors were for their middle severity rating and paradoxically both indicated decreased delays. Since the primary objective of this study was to compare the diagnostic power of the methods, we compare that for ETDRS, ETDRScen, Tissue-Damage and Metabolic factors below.

**Table 3:**
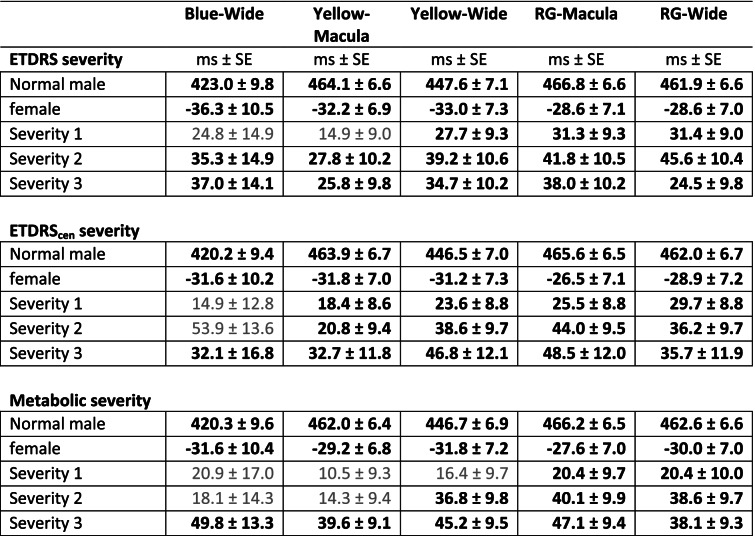
Summary of multiple linear regression models of mfPOP response delays for each stimulus protocol against disease severity ratings based on ETDRS and PCA factor 1 (Metabolic) scores, corrected for age and sex

The input data were the mean of the 44 delays per eye. Significant results are in bold, non-significant grey

**Table 4:**
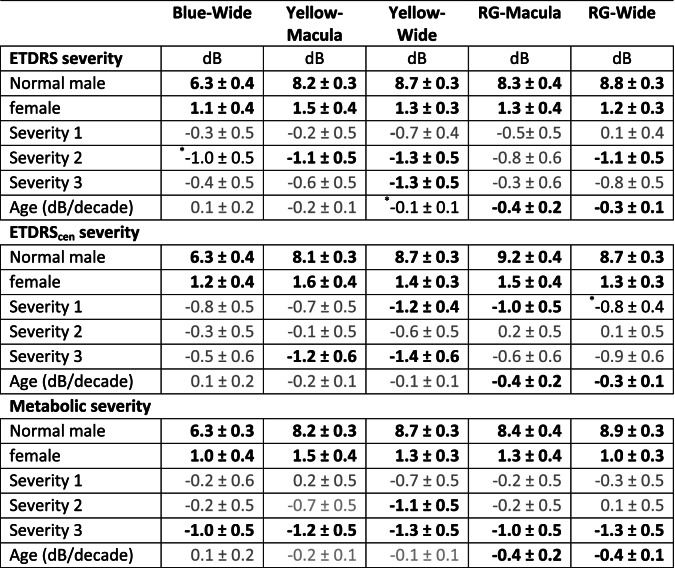
Summary of multiple linear regression models of mfPOP sensitivities against disease severity ratings based on ETDRS and PCA factor 1 (Metabolic) scores, corrected for age and sex

Significant in bold, marginal in normal text and ^*^, non-significant in grey. The input data were means of the lowest 22 of the sensitivities per eye

Table 4 provides results of similar models for decibel sensitivity for each eye. Here the (infrequent) significant values are in bold (*p* < 0.05), and non-significant figures are in grey. Marginally significant values (*p* > 0.05 and < 0.08) are in plain text and are marked with an^*^. Negative values indicate suppressed sensitivity. As for delays, the mean R^2^ increased from the ETDRS to the Metabolic factor models, rising from 0.220 to 0.282. In general response suppression increased with increasing severity ratings. The model for the Metabolic factor severity ratings had the largest number of significant coefficients, six. Yellow-Wide was perhaps the best performing protocol, with 2 severities being significant for each severity type. The blue protocol performed worst, having R^2^-values < 0.180 for all protocols. No significant results were obtained for the Tissue-Damage or Lipid factors.

To reduce the effects of multiple comparisons the models of Tables 4 and 5 were based on means of mfPOP functional performance of each eye. Previous mfPOP studies of T2D indicated that a few severely affected regions can be highly diagnostic in diabetes [17, 18]. We explored this using ROC analysis, the inputs for which were deviations from the normative data at each point in the visual field of each eye.

**Table 5:** AUC for discriminating No DR vs. mild to moderate DR in eyes classed as Metabolic severity rating 3

Test Protocol	Sensitivity	Delay	Combined	MdRank	MxRank
**Blue Wide**	76.7 ± 12.5	60.3 ± 21.4	82.1 ± 8.70	3	2
**Yellow Macula**	64.7 ± 12.2	78.7 ± 10.1	75.3 ± 10.4	5	5
**Yellow Wide**	85.9 ± 8.80	79.0 ± 9.30	80.9 ± 8.60	1	1
**RG Macula**	73.7 ± 12.7	78.5 ± 11.5	79.1 ± 11.1	2	4
**RG Wide**	76.1 ± 13.2	76.0 ± 11.8	81.5 ± 10.5	4	3

AUC values for Sensitivity, Delay and Combined scores (Methods) are given. MdRank and MxRank are the rank order of performance by the median or maximum across the 3 measures. The values are the means for the 6 to 10 *N* worst regions in the field, because these generally performed better than *N* < 6 and > 10. The SE are the RMS of the 5 individual SE values

We generated 20 ROC plots of sensitivity (true-positive rate) on the false-positive rate for sensitivities and delays for each protocol for each of the three-level disease severity ratings. The first to 20th ROC plots were based on the number (N) of the worst deviations from normal used, where *N* = 1 is the single worst deviation from normal in each field, *N* = 2 is the mean of the worst two deviations, and so on up to the mean of the worst 20 deviations. This simple method provides unbiased insights into the reliability of small numbers of deviating points.

**Fig. 2:**
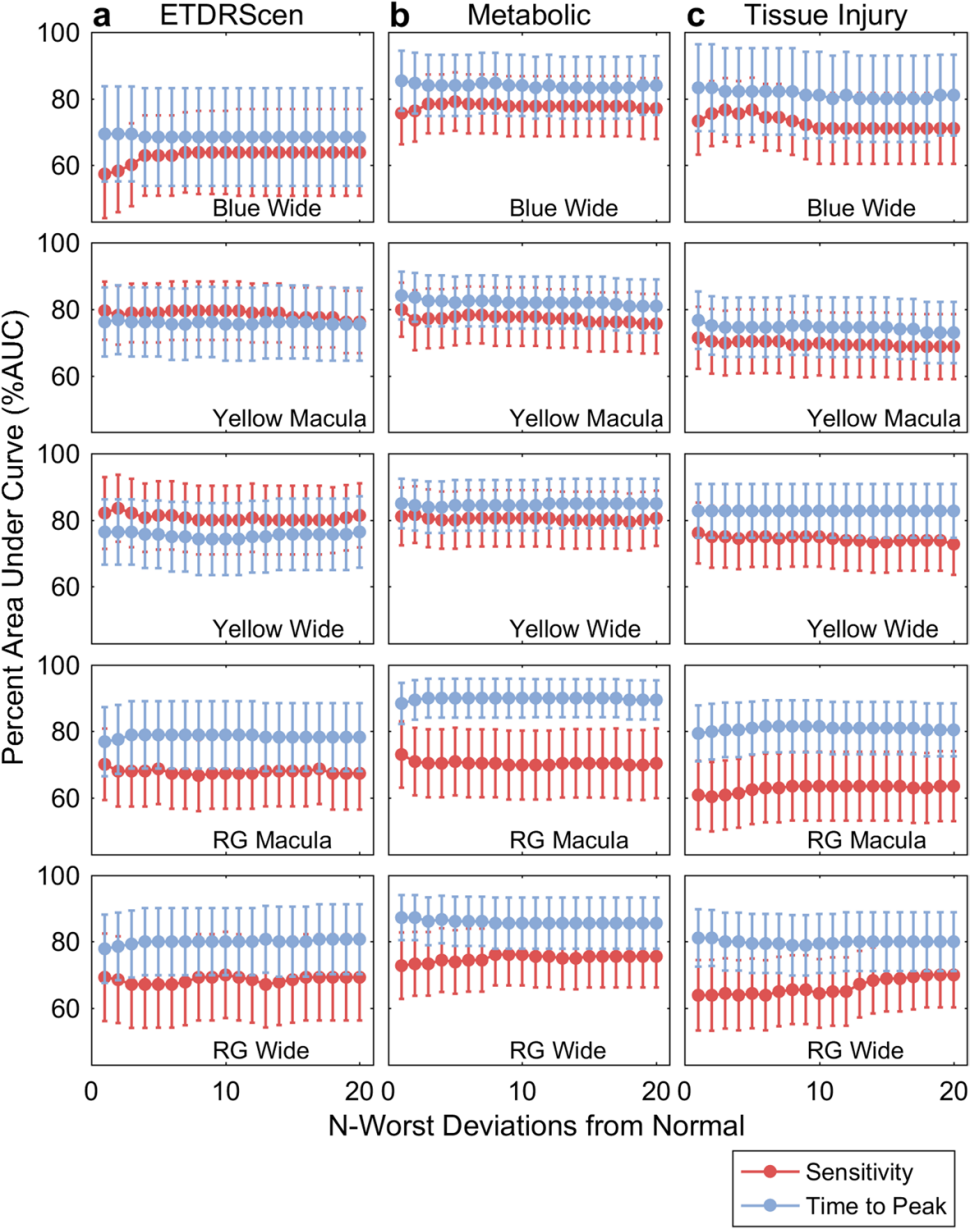
Percent Areas under ROC plots (AUCs ± SE) for the mean of *N* worst amplitude and time to peak deviations for the five protocols (rows). Discrimination was between the eyes of T1D subjects in the highest of three severity categories of Column **a**) ETDRScen; Column **b**) the Metabolic factor; and Column **c**) the Tissue-injury factor and normal control eyes. For most protocols the best diagnostic power was achieved for the first few worst regions. For ETDRScen the eyes were classed as moderate non-proliferative diabetic retinopathy, Levels 35 and 43. For the Metabolic and Tissue-injury severities the included eyes could range from ETDRS 10 to 43

The panels of Fig. 2 illustrate the results of such calculations for discriminating normal control eyes from T1D eyes, comparing control subjects and eyes in the most severe disease rating as determined by: ETDRS_cen_ scores (left column, Fig. 2a), the Metabolic scores (middle column, Fig. 2a), and the Tissue-injury scores (right column, Fig. 2c). Across protocols the Metabolic ratings gave the largest number of high AUC values for both sensitivities and delays. *N* worst deviations in the range 1 to 3 generally gave the best performance. All protocols appeared to be discriminatory for the Metabolic factor, although green on red appeared best (Fig. 2b). Blue on blue was the worst with respect to detecting retinopathy (Fig. 2a).

**Fig. 3:**
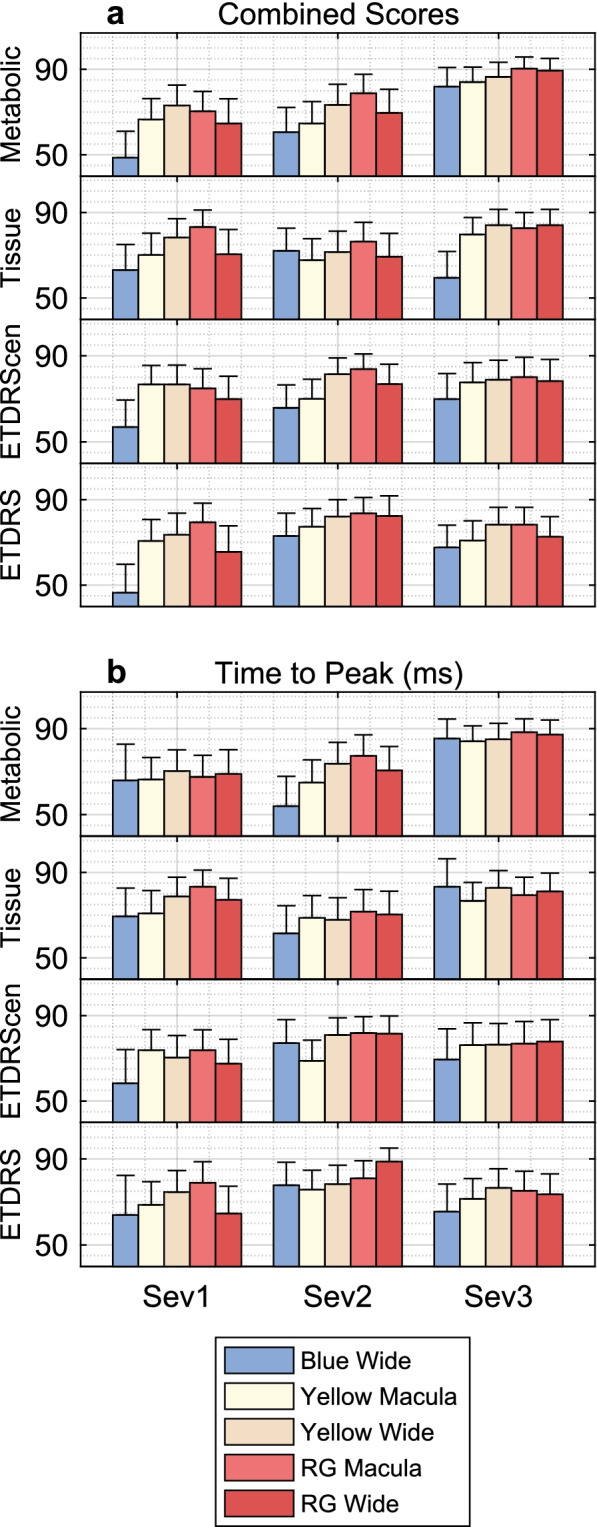
Each bar is the ROC values for the mean of the single worst region for each of the T1D eyes and control eyes, i.e. the first point in plots like those in Fig. 2. The error bars are the SE. The performance of the 5 protocols (legend) to discriminate between eyes of T1D subjects in each of the three severity ratings (Sev1 to Sev3) of the Metabolic scores, Tissue-injury scores, ETDRS_cen_, and ETDRS (rows) and normal control eyes. Panel (**a**) gives the outcomes for the combined sensitivity and delay scores (Methods), (**b**) is delay data as in Fig. 2. Generally, the Metabolic scores were best at segregating eyes with minor damage from those with more severe functional change from control eyes, i.e. the AUC values

Since there are a large number of such plots, we sought to summarise the outcomes by reducing each to one summary AUC value by taking the value for the single worst region (*N* worst =1 in Fig. 2). Figure 3 shows all results for the Metabolic and Tissue-injury PCA factors and the ETDRScen and ETDRS scores. Data are presented for delays, and the somewhat better performing combined sensitivity and delay scores (Methods). In order to compare AUC for the patient-wise PCA factors and the eye-wise ETDRS scores, we combined the latter by taking the maximum score between eyes for an individual. Thus, Severity 1 for ETDRS and ETDRScen corresponds to both eyes having a normal appearing fundus. Severity 2 represents T1D eyes with ETDRS and ETDRScen grading of no retinopathy and Severity 3 represents eyes with retinopathy.

Outcomes for the Metabolic ratings were possibly more concordant with those for ETDRS_cen_ than with ETDRS. As compared to the results for prediction of the different severities of the Tissue-injury and ETDRS scores, functional assessment as assessed by mfPOP using combined sensitivity and delay scores most strongly predicted subjects in the most severe Metabolic factor category, achieving AUCS over 90%, over the least severe category (Fig. 3a). Of note, greater change in AUCs across severity categories for predicting the Metabolic Factor, ETDRScen and ETDRS scores was evident with the blue on blue stimuli (Fig. 3a). Similar findings were evident using the mfPOP delays only, but with less evident change in AUCs across severity categories using the blue stimuli (Fig. 3b). None of the mfPOP protocols were able to better separate T1D subjects with the most severe Tissue-factor score from those with the least severity category from control subjects.

An important issue for prognostication is how well mfPOP distinguishes patients with normal fundus appearance from those with mild to moderate retinopathy. We did ROC analysis discriminating these patients, repeating it for the ETDRS, ETDRScen, Metabolic and Tissue-injury severity ratings. The Metabolic biomarker produced the best discrimination and the data are given in Table 5. The tabulated values are for the mean of the AUCs for the 6 to 10 worst regions in the field, for Metabolic severity rating 3 (Sev3). The Wide-field protocols seemed to perform better than macular ones, Yellow Wide the best overall producing AUC values across all three methods of ≥79%.

## Discussion

Some of the stimulus protocols were motivated by previous results, e.g. red-green stimuli for retinal ganglion cell death [21], which has been reported in early DR [27]; and yellow wide-field [17, 18] and macular stimuli in early DR [18] and AMD [12, 13]. Reports that diabetes damages short-wavelength cones motivated the transient blue stimuli [28]. The transient blue stimuli do not drive the slowly responding melanopsin-containing retinal ganglion cells [29, 30].

The purpose of this study was to measure the effects of T1D upon retinal function in terms of relative diagnostic power of the five methods. Unique to this study is the influence of 16 patient variables on localised retinal function. The Metabolic Biomarker was best able to segregate controls from patients (Tables 3 and 4, and Fig. 3), and also patients with normal fundi from those with mild to moderate DR (Table 5 and Fig. 3 cf. Sev1 and Sev2/3). The other two potential biomarkers performed less well in ROC analysis (Figs. 2 and 3). Combining per-region sensitivity and delay data provided the best diagnostic power (Fig. 3a, Table 5), as we have reported for T2D [17]. We have also reported that wide-field stimuli outperformed macular stimuli in early-stage T2D [18], in agreement with the findings here for the Yellow and RG protocols. That being said the RG Macular stimulus performed well (Figs. 2 and 3). The Metabolic Biomarker was correlated with HbA1c (Table 2) and in a previous study ranking the severity of the T2D patients by severity of their HbA1c levels produced good correlation between AUC achieved and HbA1c levels [18].

We have previously reported AUCs for discriminating T2D patients and controls 87.1 ± 6.3%. Here the result was more modest at 76.7 ± 8.8 for classification of severity by ETDRScen (Fig. 3 Sev1). In both cases this was for persons with two normal appearing eyes. Classification by eye, where a fellow eye could be worse, produced AUCs about 2% higher. Aside from our mfPOP studies of diabetic eye disease [17–19], other functional measures indicate that the retinal neuropathy can precede classical diabetic retinopathy [28, 31–35]. Here we found mfPOP discriminated patients with and without DR, the Yellow Wide protocol achieving AUCs between 79.0 and 85.9% (Table 5). In a clinical setting with a high prior probability of encountering a patient with retinal damage this level of diagnostic power might be useful to indicate which patients should be treated with Fenofibrate or other interventions.

AUC values in our previous studies [17, 18] were not significantly different to those reported here. In those studies, asymmetry between eyes produced significantly higher AUC values. We did not see that here and have not reported on asymmetries due to lack of space. This might suggest a difference between type 1 and type 2 diabetes eyes. Alternatively, the results might be a property of the particular subject groups.

The effect pupillary autonomic neuropathy is relatively minor because frequently the diagnostic power is carried by the few most deviating points in a field, as we have shown before [17–19], and in Fig. 2. Obviously neuropathy of the pupil cannot change the sensitivity or delay of just a few parts of the visual field. Pupil neuropathy would mimic global effects. At the request of a reviewer we examined the correlation between eyes of the regional data. In fact larger and smaller than normal sensitivities or delays can be seen frequently in the same retina [19]. We calculated the correlation between the 1672 to 2200 field points for each of the two eyes. Anatomically equivalent naso-temporal locations in the two eyes were compared. Across the 5 protocols (order as in Table 5) the values for sensitivities were: 0.421, 0.519, 0.476, 0.481, 0.487, and delays: 0.658, 0.487, 0.502, 0.489, 0.498. The low correlations go to illustrate the relative independence of regional values in the two eyes.

The blue stimulus was among the worst performing. Much work on pupillography has focused on blue stimuli with flash durations around a second (reviewed, [29, 30]). This is because things like the steady-state diameter of the pupils are driven by the very slow melanopsin driven responses of special retinal ganglion cells. We have created slow mfPOP designed to drive these cells [29, 30]. Like the faster blue stimuli used here they had lower diagnostic power than yellow stimuli. We have shown that transient blue-containing mfPOP stimuli like those used here, are more affected by changes in visual attention than yellow stimuli [36]. Blue stimuli are also more likely to be affected by lens brunescence and light scattering.

Although the results here were promising we have recently introduced a new mfPOP stimulus variant called *clustered-voll*eys that outperforms stimuli like those used here. The first demonstration was in early macular degeneration [37]. Another report on 6 studies of 96 normal subjects showed that the signal to noise ratios for the clustered-volleys method was between 35 and 57% larger than methods like those here (*p* < 0.001 in 5/6 studies) (Carle et al. Clustered volleys stimulus presentation for multifocal pupil perimetry, In revision). Using those stimuli we have compared mfPOP with structural data from optical coherence tomography and macular perimetry in T2D patients with and without mild macular oedema [19], and have shown that mfPOP functional change over time correlates well with structural change (Sabeti et al. Objective Perimetry identifies functional progression and recovery in mild diabetic macular oedema, In revision).

Published investigations of retinal function in diabetes have utilized only ETDRS-like measures and blood glucose control [38–42]. To our knowledge, this is the first report on retinal function in T1D and its association with tissue-damage and metabolic patient variables and moderate ETDRS scores. Measuring visual function in combination with Biomarkers derived from such patient data may be useful in assessing the efficacy of new treatments aimed at early stages of DR, and identifying eyes at risk of progression of retinopathy. Longitudinal studies will be required to confirm this. The Metabolic and Tissue-Damage factors are mutually uncorrelated summary measures from the patients that may reflect independent tissue-damage processes. The validity of these Biomarkers will require further study to determine if in larger cohorts the form of these markers are stable. In addition to the PCA based factors we also checked if non-orthogonal factors might explain the patient variables better, they did not. The PCA method has the advantage that the major independent sources of variation in the data are identified in an assumption free manner. Future studies with more subjects should re-examine the PCA factors and scores, and that information could underpin the creation of models for more formal severity markers. Table 2 is a start on examining what variables should be included in such models, which would likely include the effects of age and sex, and possibly the type of diabetes.

Limitations of our study were the relatively long duration of T1D (23.3–24.7 years) and the sample size for each retinopathy severity. Also, we did not take blood samples from control subjects. Finally repeatability was not investigated here, however we have shown good repeatability previously in T2D [17]. Using more modern mfPOP methods on 40 glaucoma patients and 95 match controls test-retest variability was half that of standard automated perimetry [43]. Further investigations of whether improvements in retinal function measured by mfPOP can be achieved by improving long-term blood glucose levels are warranted.

## Data Availability

The datasets used and analysed in the current study are available from the corresponding author on reasonable request due to confidentially requirements on patent applications pending.

## Abbreviations

AMD: Age-related macular degeneration
AGE: Advanced glycation end-products
Anti-VEGF: Anti-vascular endothelial growth factor
BGL: Blood glucose level
BMI: Body mass index
DR: Diabetic retinopathy
ETDRS: Early treatment of diabetic retinopathy study
GDTI: Generalised diabetes-related tissue injury
mfPOP: Multifocal pupillographic objective perimetry
mfVEPs: Multifocal visual evoked potentials
PD: Pattern deviations
PCA: Principle component analysis
T1D: Type 1 diabetes
T2D: Type 2 diabetes
